# Comparative Analysis of Clinical Severity and Outcomes in Penetrating Versus Blunt Traumatic Brain Injury Propensity Matched Cohorts

**DOI:** 10.1089/neur.2024.0009

**Published:** 2024-04-03

**Authors:** Ali Mansour, Plamena P. Powla, Ronald Alvarado-Dyer, Farima Fakhri, Paramita Das, Peleg Horowitz, Fernando D. Goldenberg, Christos Lazaridis

**Affiliations:** ^1^ Department of Neurology, Division of Neurocritical Care, University of Chicago Medical Center, Chicago, Illinois, USA.; ^2^ Department of Neurological Surgery, University of Chicago Medical Center, Chicago, Illinois, USA.; ^3^ Department of Neurology, Neurosciences Intensive Care Unit, OU Health University of Oklahoma Medical Center, Oklahoma City, Oklahoma, USA.

**Keywords:** blunt traumatic brain injury, comparative analysis, penetrating traumatic brain injury

## Abstract

Traumatic brain injury (TBI) is a global health challenge; however, penetrating brain injury (PBI) remains under-represented in evidence-based knowledge and research efforts. This study utilized data from the Trauma Quality Improvement Program (TQIP) of the National Trauma Data Bank (NTDB) to investigate outcomes of PBI as compared with clinical-severity-matched non-penetrating or blunt TBI. A total of 1765 patients with PBI were 1:1 propensity score-matched for clinical severity with blunt TBI patients. The intent of PBI was self-inflicted in 34.1% of the cases, and the mechanism was firearm-inflicted in 89.1%. Mortality was found to be significantly more common in PBI than in the severity- matched TBI cohort (33.9% vs. 14.3 %, *p* < 0.001) as was unfavorable outcome. Mortality was mediated by withdrawal of life-sustaining therapies (WOLST) 30% of the time, and WOLST occurred earlier (median 3 days vs. 6 days, *p* < 0.001) in PBI. Increased rate of mortality was observed with a Glasgow Coma Scale (GCS) of <11 in PBI as compared with <7 in blunt TBI. In conclusion, PBI patients exhibited higher mortality rates and unfavorable outcomes; one third of excess mortality was mediated by WOLST. The study also brings into question the applicability of the conventional TBI classification, based on GCS, in PBI. We emphasize the need to address the observed disparities and better understand the distinctive characteristics and mechanisms underlying PBI outcomes to improve patient care and reduce mortality.

## Introduction

Traumatic brain injury (TBI) remains a significant global health challenge, with an annual incidence affecting tens of millions of people and associated costs that soar to $400 billion.^1^ Although extensive research has been conducted to address TBI, it predominantly pertains to non-penetrating brain injuries, leaving penetrating brain injury (PBI) severely under-represented or entirely excluded. This is particularly striking given the considerable public health implications associated with PBI, mainly firearm-inflicted brain injuries. Almost half of all TBI-related deaths from recent years resulted from suicide and homicide,^2,3^ with specific demographic groups, such as young men in metropolitan areas and non-Hispanic black individuals, bearing a disproportionately higher burden. Despite the dire epidemiological data, there is a significant deficit of good quality literature on PBI. This is accompanied by a lack of high-fidelity data, databases, or standardized treatment algorithms.

The relative paucity of literature and guidelines in PBI may lead to ambiguity in regards to what may constitute an acceptable standard for desired outcomes in this patient population. Meanwhile, emerging literature from military cohorts demonstrates that a shift toward more aggressive neurosurgical and critical care strategies may significantly improve survival rates.^4,5^ This association of targeted aggressive care with decreased mortality has recently been documented in the civilian context via two comparative effectiveness research (CER) efforts based on the National Trauma Data Bank (NTDB).^6,7^

Here we leverage data from the Trauma Quality Improvement Program (TQIP) of the NTDB to explore the following questions. (1) Within clinical-severity-matched cohorts of patients facing moderate and severe TBI versus PBI, do individuals with PBI exhibit more adverse outcomes? (2) If outcome disparities exist, to what extent can these outcomes may be mediated by withdrawal of life-sustaining therapies (WOLST)? This investigation aims to shed light on the outcomes and potential reasons for disparities in the treatment and management of civilians with penetrating versus blunt TBI.

## Methods

### Data

**FIG. 1.**
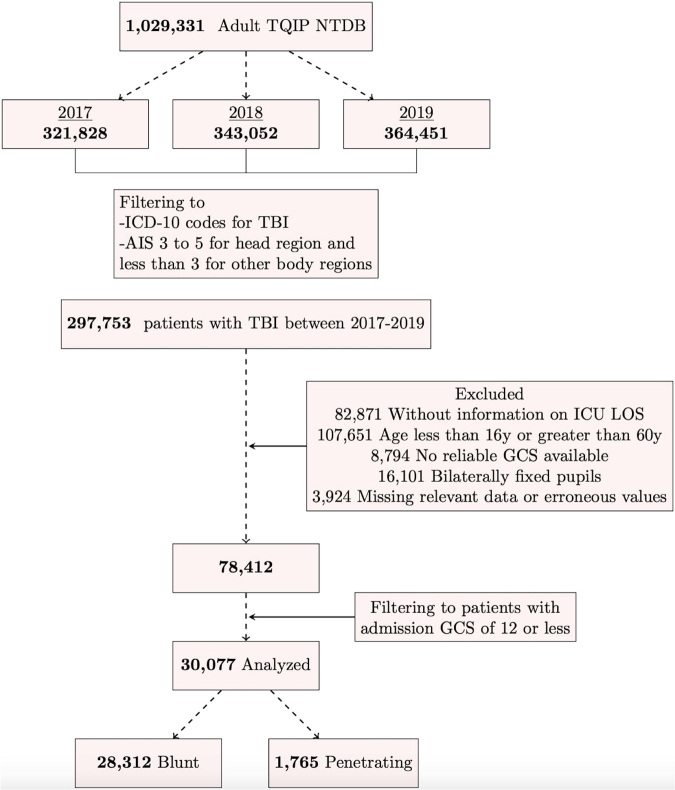
Cohort selection flow chart.

In this observational study, we employed the TQIP of the NTDB. The study was exempted from the institutional review board (IRB). Data included were collected between the years of 2017 and 2019; this time range was chosen to represent a contemporary data set including key neurological variables such as midline shift (MLS) on head computed tomography (CT) and pupillary reactivity (these variables were not recorded in prior years). Cases of PBI and blunt TBI were identified using the 10th revision of the International Statistical Classification of Diseases and Related Health Problems (ICD-10) codes for TBI and a concomitant mechanism of injury that is “penetrating” or “blunt” respectively (see Table S1). Data included were restricted to centers that participate in the TQIP, and subjects with an abbreviated injury severity (AIS) score 3–5 for the head region and <3 for other body regions. Inclusion criteria: age 16–60, Glasgow Coma Scale (GCS) on arrival of 3–12, and at least one reactive pupil on initial examination. Data were included only if key variables of interest were not missing. These variables included: outcomes of interest (hospital disposition, mortality, or WOLST status), clinical variables of interest (systolic blood pressure [SBP; also excluded if <20, to avoid spurious data], pulse oximetry [excluded if <20], GCS, and data on MLS, pupillary reactivity, or trauma type). Accordingly, a total of 30,077 patients were included in the analysis (28,313 [94.1%] blunt, and 1765 [5.9%] penetrating). See Figure 1 for flow chart describing data abstraction.

### Interventions, predictors, and outcome variables definition

Neurosurgical intervention was defined as an open approach cranial procedure involving release, drainage, or extirpation of brain matter and was coded through the ICD-10 Procedure Coding System (ICD-10-PCS) (see Table S2). It is of note that these codes do not overlap with the code for external ventricular drain (EVD) insertion which is typically coded as ICD-10-PCS 009630Z: Drainage of Cerebral Ventricle with Drainage Device, Percutaneous Approach.

Intracranial pressure (ICP) monitor placement was a variable included in the data set as was identified accordingly. ICP monitoring status was considered present if the patient had a parenchymal probe intended for ICP monitoring or an EVD.

Other variables included age, initial SBP, initial pulse oximetry, first recorded GCS (within 30 min or less of arrival), first recorded GCS-motor (within 30 min or less of arrival), Injury Severity Score (ISS), MLS >5 mm within 24 h of injury, and pupillary reactivity (within 30 min or less of patient arrival). The selection of these variables follows Haider and coworkers' recommendations when studying trauma- related mortality and adds to them important neurological features necessary to predict mortality based on the International Mission for Prognosis and Analysis of Clinical Trials (IMPACT core),^8^ as well as important imaging predictors such as MLS.

Outcomes of interest were mortality, rate of WOLST, and dispositional outcome as defined by hospital discharge location. Mortality was defined as death by the time of hospital discharge, WOSLT, or discharge to a hospice facility. Because the NTDB does not include data on long-term outcomes, we defined unfavorable outcome as death or discharge disposition to a long-term acute care hospital (LTACH), or a skilled nursing facility (SNF). Meanwhile favorable outcome was leaving against medical advice, transfer to court/law enforcement or a psychiatric hospital, or discharge to one of the following: home, a short-term general hospital for inpatient care, an intermediate care facility, or an acute rehabilitation facility.

### Statistical analysis

#### Propensity score (PS) matching

The data set was divided into two groups based on the mechanism of injury (blunt vs. penetrating). Statistical analyses were conducted using parametric testing (unpaired *t* test) for continuous variables and Pearson χ^2^ test for categorical variables. *P* value of <0.05 was considered statistically significant. Standardized mean difference (SMD) was used to examine the balance of covariate distribution between the two groups (blunt vs. penetrating). We used PS matching to create a subgroup of patients within which covariates of interest are represented in a balanced manner, therefore reducing the bias in estimates of outcome. To calculate the PS, a logistical regression was performed to define the odds ratio (OR) for each variable associated with the mechanism of injury. The variables used included: age, initial GCS-M, initial SBP, initial pulse oximetry, presence of MLS, ISS, and pupillary reactivity. We utilized the “MatchIt” library in R studio (version 2022.07.2, © 2009–2022 RStudio) to match the data; specifically, we used: nearest neighbor matching (NNM), as it yielded the most favorable matching result. Matching was performed on a 1:1 basis. Within the matched cohort, the association between the mechanism of injury and mortality, dispositional outcome, and rate of WOLST was calculated via a logistical regression with 1765 patients in each group.

#### Mediation analysis

To explore the relationship among mechanism of injury, mortality and WOLST we performed a statistical mediation analysis. In mediation analysis, the effect of a baseline condition (mechanism of injury) on an outcome (mortality) is separated into direct and indirect effects. The indirect effect may be explained through a mediator (WOLST in this case). Mediation analysis is here used to quantify the effect of WOLST on excess mortality.^9^ For that purpose, we utilized the “Mediation” library in R studio (version 2022.07.2, © 2009–2022 RStudio).

#### Excluding WOLST

In a separate analysis, to further explore the relationship between mechanism of injury and mortality, we excluded any patient who underwent WOLST from the original data set, and performed the matching on data that did not include any WOLST. Similarly, a logistical regression was used to explore the relationship between mechanism of injury and outcomes of interest (mortality and dispositional outcome) in this data set.

#### IMPACT core evaluation

**FIG. 2.**
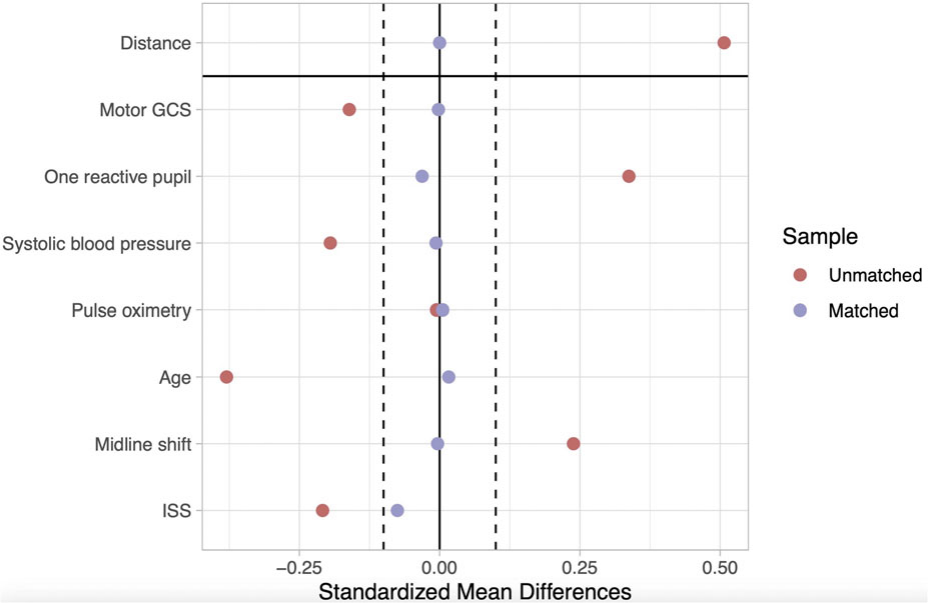
Covariate standardized mean difference (SMD) before and after matching.

**FIG. 3.**
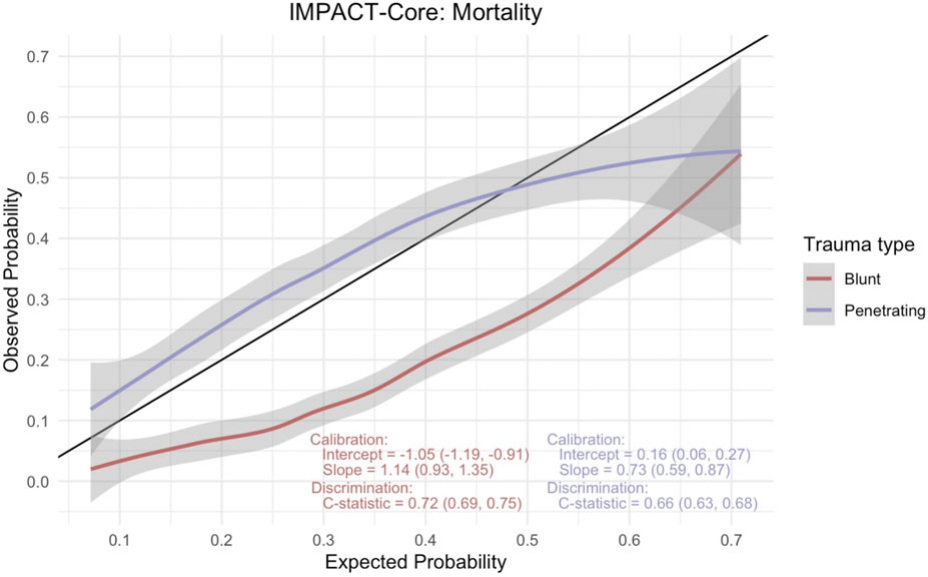
International Mission for Prognosis and Analysis of Clinical Trials (IMPACT)-core mortality calibration curve.

The IMPACT model is a series of prognostic models of increasing complexity that incorporate admission characteristics to predict unfavorable outcomes at the 6-month time point following moderate or severe TBI.^8,10,11^ The core model encompasses age, GCS motor, and pupillary reactivity. We evaluated the model performance (discrimination and calibration) in predicting mortality across the two groups representing the two mechanisms of injury. Discrimination evaluates a model's ability to distinguish between patients with and without the desired outcome. The area under the receiver operating characteristic curve (AUC) serves as the metric, with 1 being the most impeccable discrimination possible and 0.5 implying that the model discriminatory ability is no better than random chance. Calibration assessment involved plotting observed versus predicted outcomes (Figs. 2 and 3). Calibration was evaluated by fitting a logistical regression model using model predictions as an offset variable; the intercept within this model indicates systematic underestimation or overestimation of predictions and should ideally be zero. The calibration slope reflects the average effects of the predictors in the model and was estimated using a logistical regression model. In a perfect model, the calibration slope equals 1.

#### GCS–mortality association

To explore the relationship between total GCS score and mortality in our cohort, and for each of the two mechanisms of interest (blunt vs. penetrating) we describe the proportion and 95% confidence interval (CI) of mortality at each GCS score for what is traditionally described as severe or moderate TBI (GCS 3–12).

#### Sensitivity analysis (E-value)

We employed sensitivity analysis to evaluate the potential influence of unmeasured or uncontrolled confounding variables and to gauge the robustness of the observed association between the mechanism of injury and mortality. In this analysis, we calculated the E-value, which represents the minimum strength of association required for an unmeasured confounder to have both with the mechanism of injury and with the outcome (mortality) to explain away the observed mechanism–outcome association, while considering the measured covariates.^12^ Our goal was to quantify the effect of possible hidden bias that might affect the results derived from the PS-matched analysis.

## Results

Of 1,029,331 unique adult TQIP encounters available in the NTDB between the years 2017 and 2019 (2017:321,828; 2018:343,052; 2019:364,451) 30,077 met inclusion criteria (2017: 10,092; 2018: 10,050; 2019: 9,935). The entire cohort comprised 94.1% blunt TBI (28,312), 22.6 % female, median age, 35 (25, 49) years (Fig. 1). A total of 5206 (14.0%) died and a total of 4363 (14.5%) had neurosurgical intervention at some point throughout their hospitalization (See Table 1). Across the 3 years, rate of PBI was relatively similar (5.8%, 6.0%, and 5.8% for 2017, 2018, and 2019 respectively, *p* = 0.7) (Table S3). The mechanism of PBI was firearm-related in 1573 patients (89.1%) with the remaining attributed to low-velocity mechanisms. The mechanism of blunt trauma was fall-related in 17,011 (60.6%) of patients (see Table S3). Patients in the penetrating group were more likely to have MLS (31.0 % vs. 20.0 % in the blunt group, *p* < 0.001). Median total GCS on presentation was 5 (interquartile range [IQR] 3–8) in the overall population; median total GCS on presentation was 5 (IQR 3–8) in the blunt TBI group and 3 (IQR 3–7) in the PBI group. A total of 21,394 blunt TBI patients had severe TBI (75.6%) compared with 1430 (81%) with PBI (Table 1).

**Table 1.** Patient Characteristics for the Unmatched Cohort

Characteristic	Overall,* n* = 30,077*^a^*	Blunt,* n* = 28,312*^a^*	Penetrating,* n* = 1765*^a^*	*p *value*^b^*
Age	35 (25.0, 49)	36 (25.0, 49)	30 (22.0, 41)	<0.001
Sex				<0.001
Female	6806 (22.6%)	6513 (23.0%)	293 (16.6%)	
Male	23,265 (77.4%)	21,794 (77.0%)	1471 (83.4%)	
Pulse	96 (80.0, 114)	96 (80.0, 114)	94 (74.0, 117)	0.005
Respiratory rate	18 (16.0, 22)	18 (16.0, 22)	18 (15.0, 22)	0.033
Pulse oximetry	99 (96.0, 100)	99 (96.0, 100)	100 (97.0, 100)	<0.001
Total GCS	5 (3, 8)	5 (3, 8)	3 (3, 7)	<0.001
Total GCS category				<0.001
Moderate	7253 (24.1%)	6,918 (24.4%)	335 (19.0%)	
Severe	22,824 (75.9%)	21,394 (75.6%)	1430 (81.0%)	
Motor GCS	2 (1, 5)	2 (1, 5)	1 (1, 4)	<0.001
WOLST				<0.001
No WOLST	27,629 (91.9%)	26,149 (92.4%)	1480 (83.9%)	
WOLST	2448 (8.1%)	2163 (7.6%)	285 (16.1%)	
Days to WOLST	7 (3.0, 11)	7 (4.0, 12)	3 (2.0, 7)	<0.001
ICU length of stay	7 (3.0, 15)	7 (3.0, 15)	7 (3.0, 14)	<0.001
Neurosurgery				<0.001
Neurosurgery	4363 (14.5%)	3816 (13.5%)	547 (31.0%)	
No neurosurgery	25,714 (85.5%)	24,496 (86.5%)	1218 (69.0%)	
Pupillary reactivity				<0.001
Both reactive	27,299 (90.8%)	25,931 (91.6%)	1368 (77.5%)	
One reactive	2778 (9.2%)	2381 (8.4%)	397 (22.5%)	
Midline shift				<0.001
Midline shift	6,199 (20.6%)	5,652 (20.0%)	547 (31.0%)	
No midline shift	23,878 (79.4%)	22,660 (80.0%)	1,218 (69.0%)	
ICP monitor	5643 (28.2%)	5234 (27.7%)	409 (35.3%)	<0.001
ISS	25 (17.0, 33)	25 (17.0, 33)	25 (18.0, 29)	<0.001
Outcome				<0.001
Favorable	19,283 (64.1%)	18,418 (65.1%)	865 (49.0%)	
Unfavorable	10,794 (35.9%)	9894 (34.9%)	900 (51.0%)	
Mortality				<0.001
Alive	25,871 (86.0%)	24,704 (87.3%)	1167 (66.1%)	
Dead	4206 (14.0%)	3608 (12.7%)	598 (33.9%)	

^a^ Median (interquartile range [IQR]); *n* (%)

^b^ Wilcoxon rank sum test; Pearson's χ^2^ test

GCS, Glasgow Coma Scale; WOLST withdrawal of life-sustaining therapies, ICU, intensive care unit; ICP, intracranial pressure; ISS, Injury Severity Score.

**FIG. 4.**
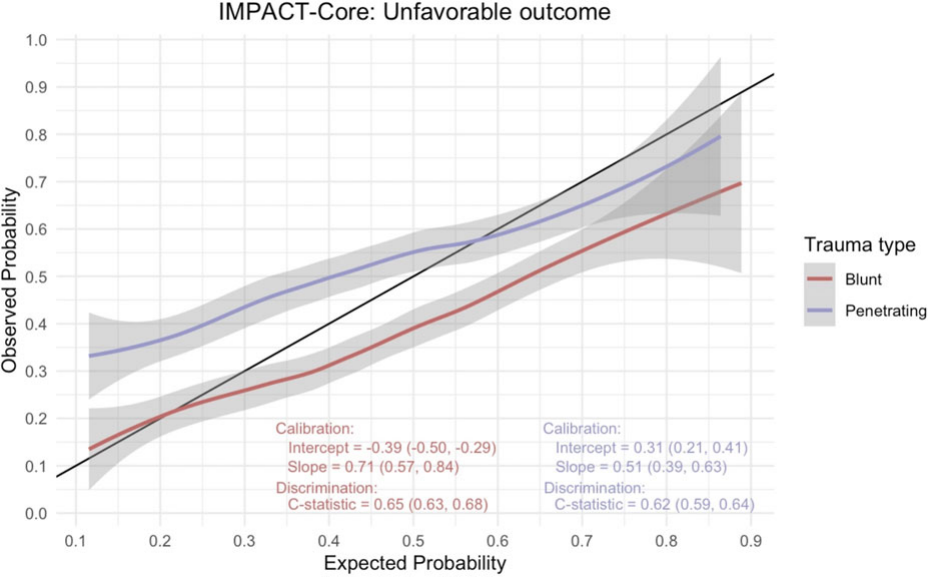
International Mission for Prognosis and Analysis of Clinical Trials (IMPACT)-core unfavorable outcome calibration curve.

Of all patients included in the analysis, 3530 were matched (1765 in each arm). Table 2 shows the variables of interest in the matched cohort. Figure 4 shows the covariate SMD before and after matching. All variables except for neurosurgery during hospitalization were within <0.1 SMD of each other (an SMD <0.1 is considered negligible).^13^ In the matched cohort (overall mortality 24.1%), 598 patients with PBI (33.9 %) died compared with 252 (14.2 %) in the blunt group. Patients who had a PBI were less likely to survive (OR 0.33; 95% CI: 0.28, 0.38, *p* < 0.001), less likely to have a favorable outcome (OR = 0.53; 95% CI: 0.46, 0.61, *p* < 0.001), and more likely to have WOLST (OR = 2.22; 95% CI: 1.79, 2.75, *p* < 0.001). The E-values for each OR calculated from the matched data set were 2.88, 2.09, and 2.34, respectively.

**Table 2.** Patient Characteristics for the Matched Cohort

Characteristic	Blunt,* n* = 1765*^a^*	Penetrating,* n* = 1765*^a^*	SMD
Age	29 (22.0, 41)	30 (22.0, 41)	-0.01
Sex			0.19
Female	428 (24.2%)	293 (16.6%)	
Male	1337 (75.8%)	1471 (83.4%)	
Pulse	94 (78.0, 113)	94 (74.0, 117)	-0.02
Respiratory rate	18 (16.0, 21)	18 (15.0, 22)	0.01
Pulse oximetry	99 (97.0, 100)	100 (97.0, 100)	-0.01
Total GCS	3 (3, 7)	3 (3, 7)	0.02
Total GCS category			0.02
Moderate	346 (19.6%)	335 (19.0%)	
Severe	1,419 (80.4%)	1,430 (81.0%)	
Motor GCS	1 (1, 4)	1 (1, 4)	0.01
ICU length of stay	7 (3.0, 14)	7 (3.0, 14)	0.00
Neurosurgery			0.31
Neurosurgery	313 (17.7%)	547 (31.0%)	
No neurosurgery	1452 (82.3%)	1218 (69.0%)	
Pupillary reactivity			0.04
Both reactive	1341 (76.0%)	1368 (77.5%)	
One reactive	424 (24.0%)	397 (22.5%)	
Midline shift			0.00
Midline shift	551 (31.2%)	547 (31.0%)	
No midline shift	1214 (68.8%)	1218 (69.0%)	
ICP monitor	383 (32.6%)	409 (35.3%)	0.06
ISS	25 (17.0, 30)	25 (18.0, 29)	0.06
Outcome			0.31
Favorable	1136 (64.4%)	865 (49.0%)	
Unfavorable	629 (35.6%)	900 (51.0%)	
Mortality			0.47
Alive	1513 (85.7%)	1167 (66.1%)	
Dead	252 (14.3%)	598 (33.9%)	

^a^ Median (interquartile range [IQR]); *n* (%)

SMD, standardized mean difference; GCS, Glasgow Coma Score; ICU, intensive care unit; ICP, intracranial pressure; ISS, Injury Severity Score

### Mediation analysis and excluding WOLST

The mediation analysis was purposed to evaluate the relationship among PBI, WOLST, and excess mortality. It showed that the indirect effect of the trauma type (PBI) on mortality via WOLST is statistically significant (ab = -0.056, 95% CI = [-0.088, -0.05]). The proportion mediated is 30.6%. This quantifies the extent to which the effect of the mechanism of injury on mortality is explained by WOLST. After excluding WOLST from the analysis, in a new matched cohort (see Table S4), PBI remained associated with decreased odds of survival (OR 0.23, CI 0.18–0.29, *p* < 0.001) and favorable outcome (OR 0.57, CI 0.49–0.66, *p* < 0.001).

### IMPACT score

We employed the impact core model to analyze a matched data set encompassing subsets of blunt and penetrating TBI. The primary outcomes examined were mortality and unfavorable disposition, at discharge with calibration curves illustrated in Figures 2 and 3, respectively. For the prediction of mortality, the AUC C-statistic for the model in the blunt TBI cohort was 0.72 (95% CI: 0.69–0.75), whereas that for PBI was lower, at 0.66 (95% CI: 0.63–0.68). Similarly, when assessing unfavorable disposition as the outcome, the C-statistic for blunt TBI was 0.65 (95% CI: 0.63–0.68), and for PBI it was 0.62 (95% CI: 0.59–0.64). Calibration curves, provided in the accompanying figures (Figs. 2 and 3), were also used to evaluate the model's performance. These calibration curves revealed notable trends in the model's predictive accuracy. Specifically, we observed that the impact core model tended to overestimate both mortality and unfavorable outcomes in cases of blunt TBI, while underestimating these outcomes in instances of PBI.

### GCS and mortality

**FIG. 5.**
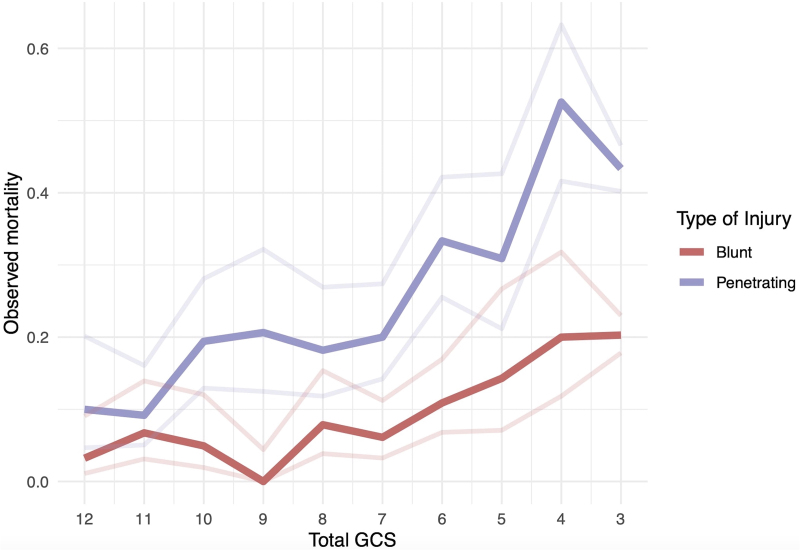
Matched Glasgow Coma Scale (GCS) and mortality percent probability.

Figure 5 demonstrates percent mortality across GCS score on presentation for blunt versus penetrating TBI. PBI is associated with higher mortality across all GCS categories. In blunt TBI, mortality progressively increases from 6.1% to 20.3% as GCS drops from 7 to 3. In PBI, mortality starts to increase as GCS drops below 11 from 9.2% to 43.4%.

## Discussion

To our knowledge, this is the first propensity-matched comparative analysis of civilian PBI versus blunt TBI cohorts. PBI is considered the most lethal form of TBI with an all-comer 70–90% mortality, and ∼50% of those who survive to reach the hospital die during hospitalization.^14,15^ Concurrently, there has been a notable absence of dedicated study to investigate the outcomes of PBI in comparison with blunt TBI patients, particularly when such comparisons are rigorously matched for neurological severity proxies such as GCS, MLS, and pupillary reactivity. Saliently, it has been demonstrated that WOLST is a common proximate cause of death in blunt TBI.^16,17^ However, the impact of WOLST in PBI mortality has not been systematically studied.^18,19^ Here we share analysis to shed light on these questions. In clinically matched cohorts of PBI versus TBI from the NTDB, PBI was associated with higher odds of mortality and unfavorable outcomes; WOLST explains approximately one third of the excess mortality in the PBI cohort, as seen by mediation analysis. Moreover, it is worth noting that WOLST occurred sooner in PBI, with a median of 3 days in contrast to 6 days after blunt TBI. These findings demonstrate and quantify the role of WOLST in explaining the higher mortality of PBI.

Having explored the role of WOLST in the higher mortality rates observed in PBI cases, it becomes important to underscore that a substantial portion of this increased mortality remains unaccounted for by WOLST. This is shown by the separate analysis excluding patients subjected to WOLST, and reinforces the increased mortality rates within the clinically severity matched PBI cohort, indicating the existence of other, as yet unexplored, factors contributing to this marked difference in outcomes. The increased rate of neurosurgical interventions in PBI (31% vs. 17.7% in the matched cohorts) suggests different pathologies, plausibly including more space-occupying hematomas requiring evacuation and performance of more decompressive craniectomies. In a recent CER study from the NTDB, we showed that neurosurgical intervention in the first 24 h, in matched PBI cohorts, was associated with improved survival.^6^ The destructive effect of high-velocity missile injuries traversing multiple lobes or injuring deep structures may further explain outcome differences. In fact, vector ballistics analysis has revealed a so-called *zona fatalis*, located ∼4 cm above the dorsum sellae, including the third ventricle, body of the corpus callosum, and cingulum.^20,21^ Bullet passage through this supra-dorsum sellar transventricular zone was universally associated with fatal outcome. Although we used MLS, pupillary reactivity, and the GCS as proxies for clinical and imaging severity, the specification of injury through imaging data remains absent within the NTDB. Further, a clear imaging classification specific to PBI is lacking when compared with blunt TBI. In addition, the unique nature and mechanisms of secondary brain injury related to PBI versus blunt TBI remain unexplored.

The IMPACT score, originally developed and validated for intermediate follow-up (at 6 months) following moderate and severe TBI, underscores an overarching concern in the scientific literature that prognostic models for TBI may be utilized by clinicians to inappropriately guide individualized clinical decisions, despite the original purpose of these models being geared toward facilitating research study design.^22^ In this case, the model exhibits inadequate discrimination for early outcome prediction, particularly in the context of PBI, further emphasizing that despite comparable clinical severity, the two disease states exhibit notable variations in outcomes. Another illustration of this variability in disease severity is manifested in the elevated mortality rates observed in PBI cases with higher GCS scores when compared with blunt TBI, thereby casting doubts on the applicability of GCS and its associated classifications (mild, moderate, and severe) in the context of PBI.

### Limitations

In addition to the limitations already mentioned, the NTDB does not encompass comprehensive data concerning long-term functional outcomes. As a result, our analysis relied on dispositional outcomes, mainly predicated on the patient's discharge location from the hospital. It is crucial to acknowledge that this serves as only a rudimentary proxy for patient outcomes, and there may be substantial disparities when compared with the more nuanced and comprehensive realm of long-term functional assessments. Our calculation of the propensity score hinged on patient characteristics available within the NTDB, and therefore, unadjusted confounding variables may still exert an influence. Imaging features, including projectile trajectory and the specific brain structures involved, carry paramount significance in such scenarios, yet regrettably, these details remain beyond the scope of our analysis and the topic of future work. We conducted a sensitivity analysis to assess the minimal strength required by an unmeasured confounding variable to nullify our observed results, as indicated by the E-values reported. Such analysis provides the strength of association that an unmeasured confounder would independently have to have with the type of injury and the outcome in order to explain away the increased OR for PBI-mortality, but weaker confounding could not do so. Finally, the IMPACT score is validated for assessing intermediate and long-term outcomes in blunt TBI.^8,22^ Here, we employ it to scrutinize immediate dispositional outcomes, as a first approximation and comparison of its distinctive performance in the context of PBI compared with blunt TBI.

## Conclusion

When matched for GCS, MLS, and pupillary reactivity, patients with PBI consistently exhibit higher mortality rates and unfavorable disposition outcomes compared with those with blunt TBI. A third of the excess PBI mortality can be attributed to the more rapid WOLST. The variability in mortality rates, particularly in PBI patients with higher GCS scores, calls into question the conventional GCS-based classification of moderate versus severe TBI as applied to PBI. A number of important factors such as missile trajectories, imaging characteristics, and mechanisms of secondary brain injury, which may explain the higher mortality in PBI, remain underexplored. Future work needs to address these in order to derive effective clinical management protocols and to inform PBI-specific research efforts.

## Supplementary Material

Supplemental data

Supplemental data

Supplemental data

Supplemental data

## Data Availability

Code to reproduce the data used will be made available through a request directed to the corresponding author.

## Abbreviations Used

AIS: abbreviated injury severity
AUC: area under the receiver operating characteristic curve
CER: comparative effectiveness research
CI: confidence interval
CT: computed tomography
EVD: external ventricular drain
GCS: Glasgow Coma Scale
ICD-10: 10th revision of the International Statistical Classification of Diseases and Related Health Problems
ICP: intracranial pressure
IMPACT: International Mission for Prognosis and Analysis of Clinical Trials
IQR: interquartile range
IRB: institutional review board
ISS: Injury Severity Score
LTACH: long-term acute care hospital
MLS: midline shift
NNM: nearest neighbor matching
NTDB: National Trauma Data Bank
OR: odds ratio
PBI: penetrating brain injury
PS: propensity score
SBP: systolic blood pressure
SMD: standardized mean difference
SNF: skilled nursing facility
TBI: traumatic brain injury
TQIP: Trauma Quality Improvement Program
WOLST: withdrawal of life-sustaining therapies
