# Supplementary figures and images for: Comparative Analysis of Clinical Severity and Outcomes in Penetrating Versus Blunt Traumatic Brain Injury Propensity Matched Cohorts
Source: Neurotrauma Rep. 2024 Apr 3;5(1):348–58. doi: 10.1089/neur.2024.0009 (PMC11002325; doi:10.1089/neur.2024.0009)

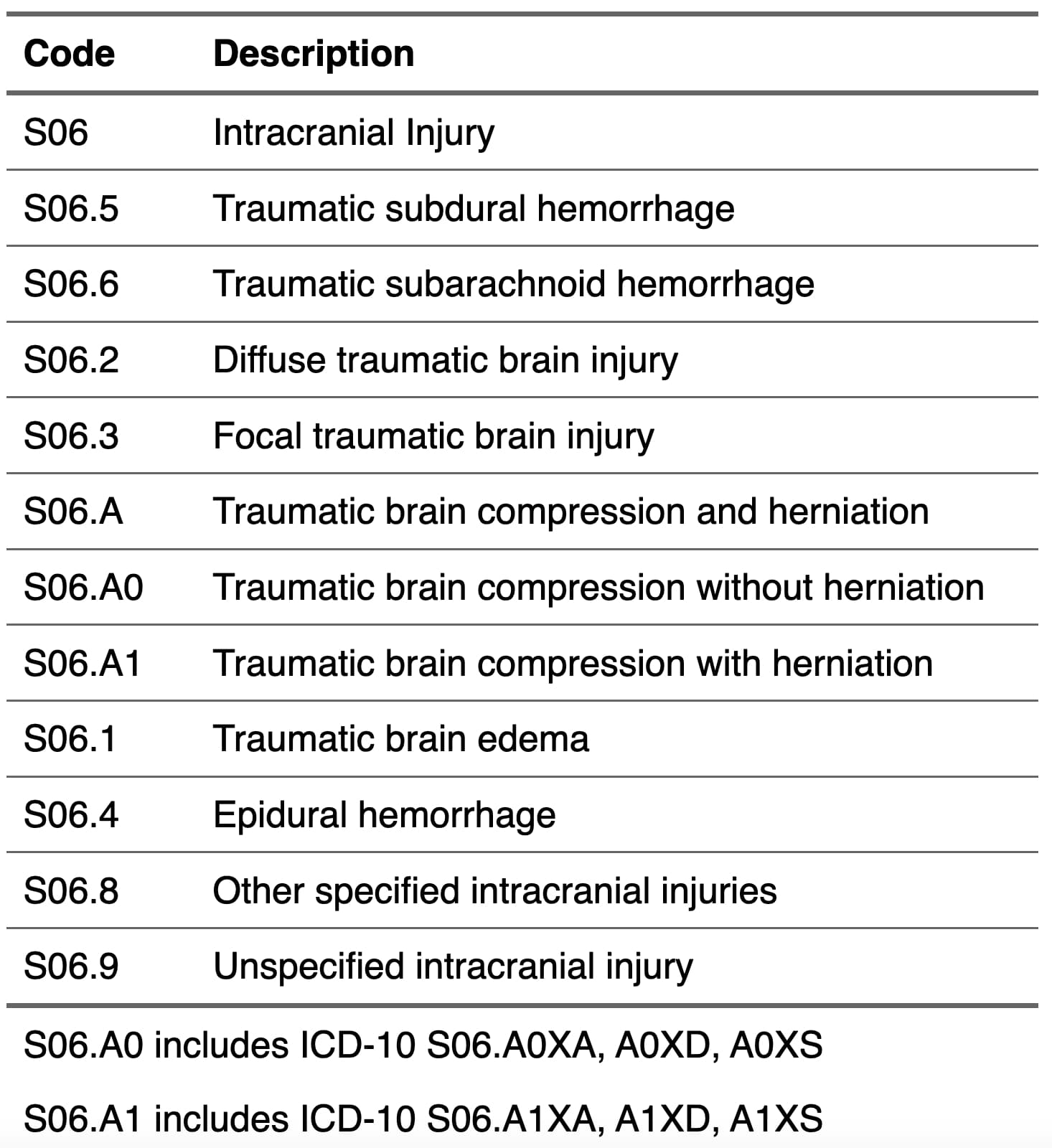

Supplement: Supplemental data [file Suppl_TableS1.doc]

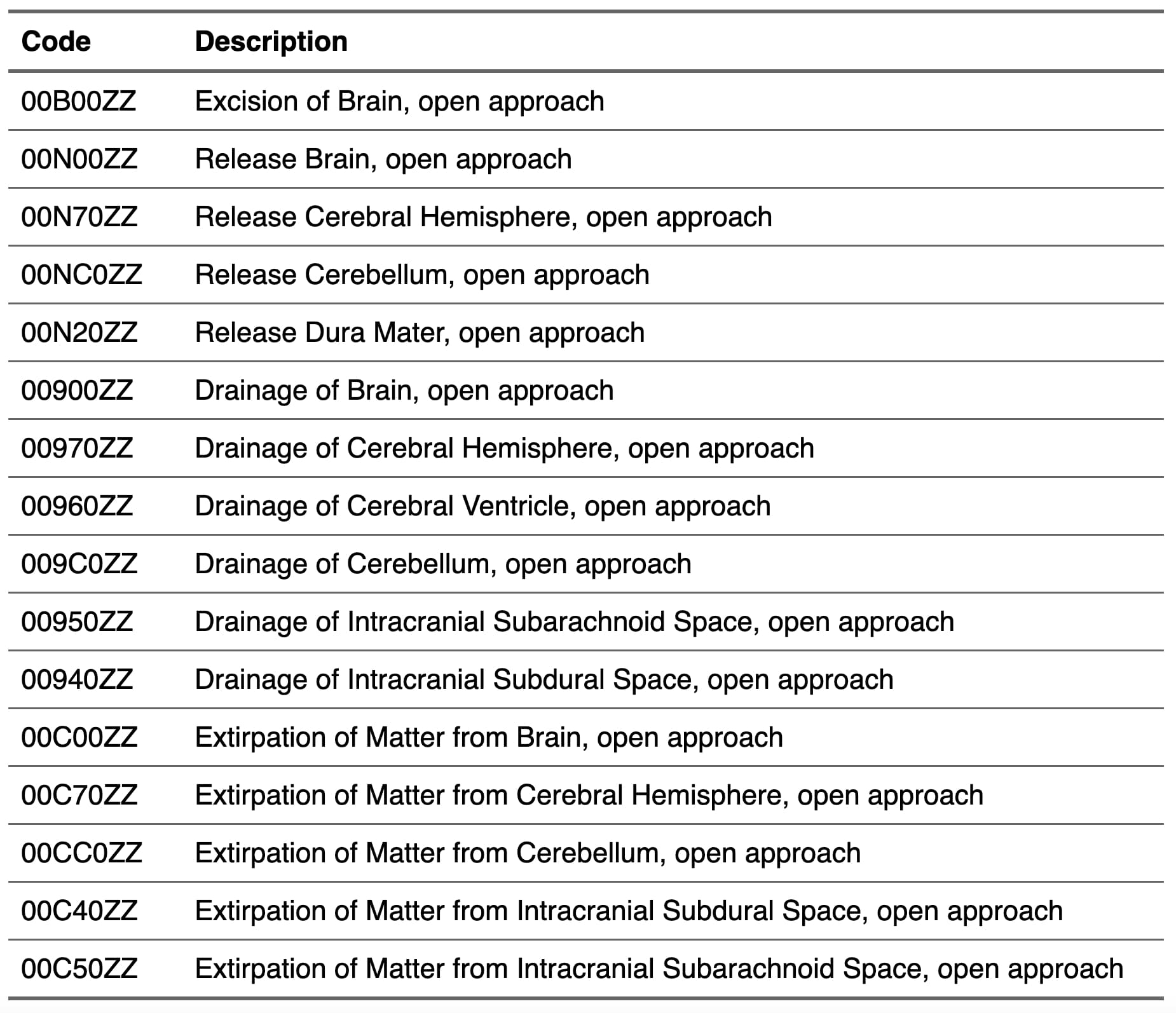

Supplement: Supplemental data [file Suppl_TableS2.doc]

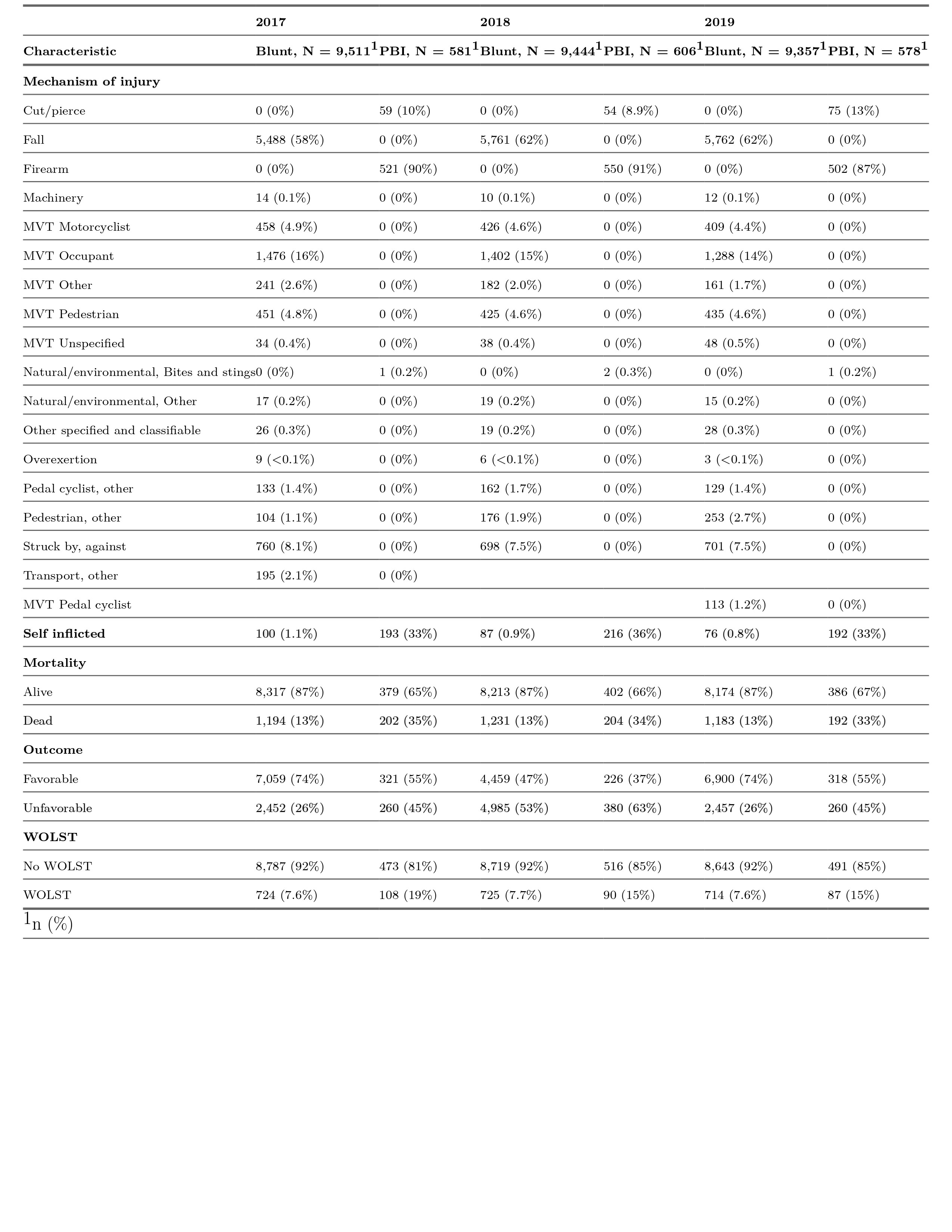

Supplement: Supplemental data [file Suppl_TableS3.doc]

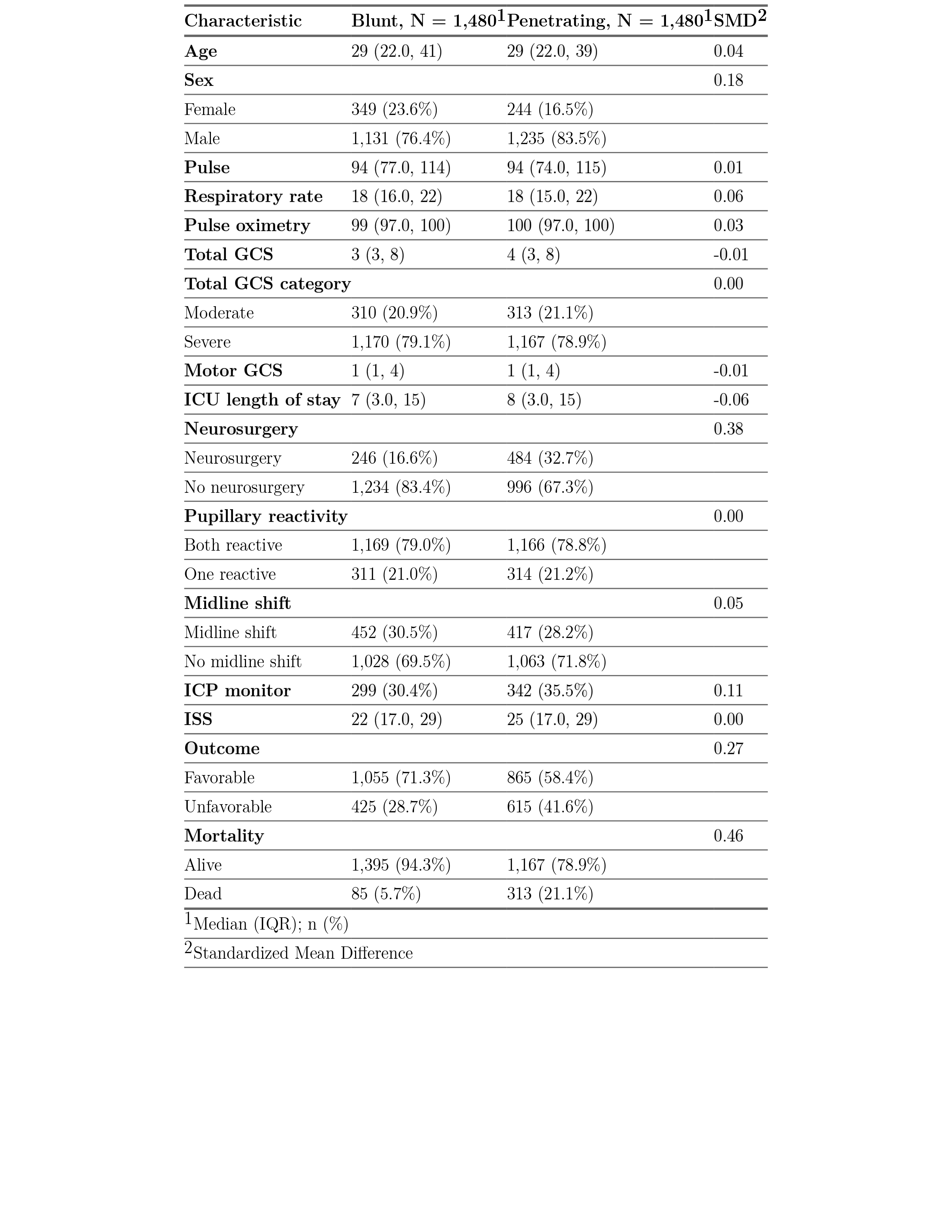

Supplement: Supplemental data [file Suppl_TableS4.doc]
